# Supplementary material for: Prox1 Suppresses the Proliferation of Breast Cancer Cells via Direct Inhibition of c-Myc Gene Expression
Source: Cells. 2023 Jul 17;12(14):1869. doi: 10.3390/cells12141869 (PMC10377922; doi:10.3390/cells12141869)
Supplement: Supplementary file 1 [file cells-12-01869-s001.zip › cells-2501066-supplementary.pdf]

Supplementary Figures and Figure Legends

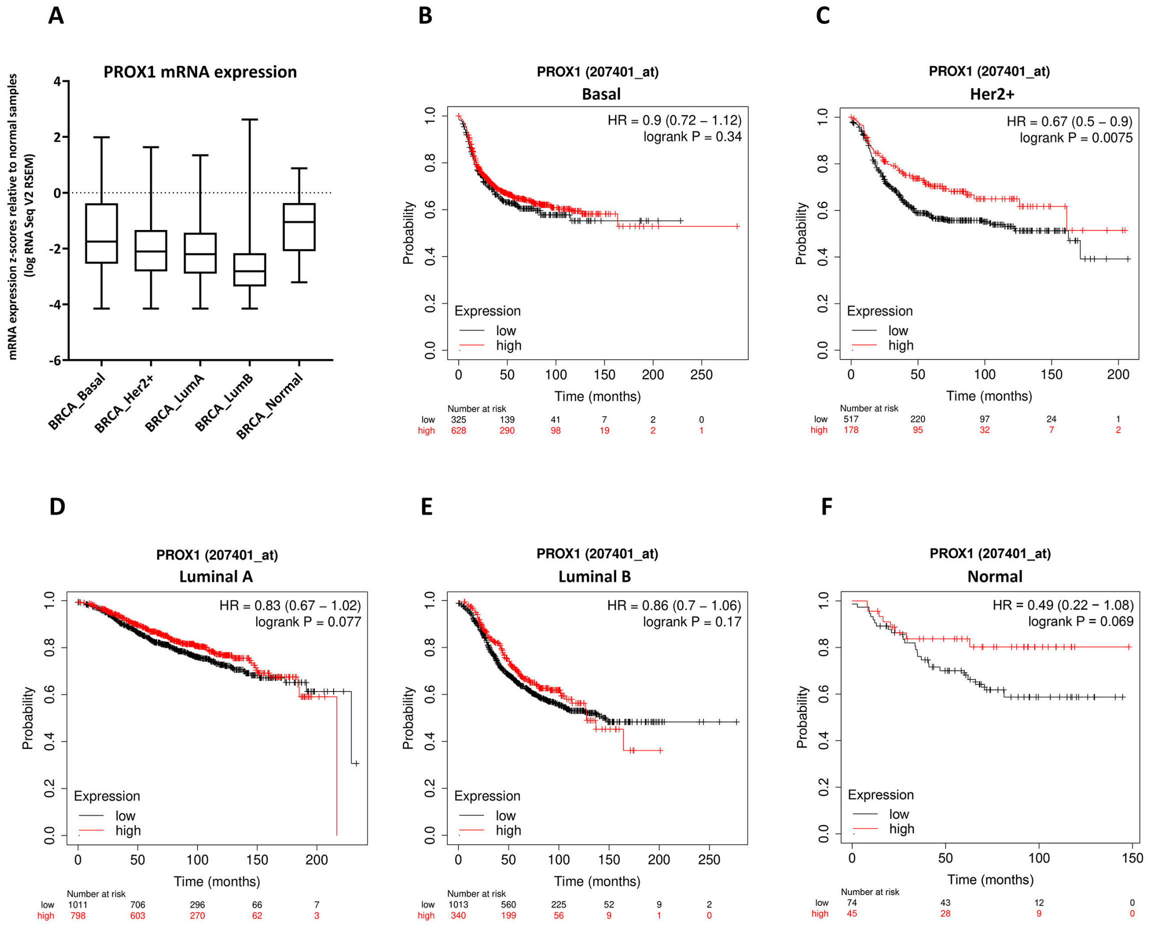

Supplementary Figure S1. The expression of Prox1 is reduced in various subtypes of breast tumors. (A) Expression of Prox1 mRNA in various breast cancer subtypes (basal, her2+, luminal A, luminal B, and normal-like) normalized with healthy tissue samples. These data were obtained from the cBioPortal database and based on the data from Breast Invasive Carcinoma (TCGA, PanCancer Atlas) study. (B) Survival curve (Kaplan-Meier) of breast cancer patients (basal subtype) with relative high (red) and low (black) expression of Prox1

from the KM-plotter (https://kmplot.com), (C) Survival curve (Kaplan-Meier) of breast cancer patients (Her2+ subtype) with relative high (red) and low (black) expression of Prox1 from the KM-plotter (https://kmplot.com), (D) Survival curve (Kaplan-Meier) of breast cancer patients (Luminal A subtype) with relative high (red) and low (black) expression of Prox1 from the KM-plotter, (E) Survival curve (Kaplan-Meier) of breast cancer patients (Luminal B subtype) with relative high (red) and low (black) expression of Prox1 from the KM-plotter, (F) Survival curve (Kaplan-Meier) of breast cancer patients (Normal-like subtype) with relative high (red) and low (black) expression of Prox1 from the KM-plotter.

A

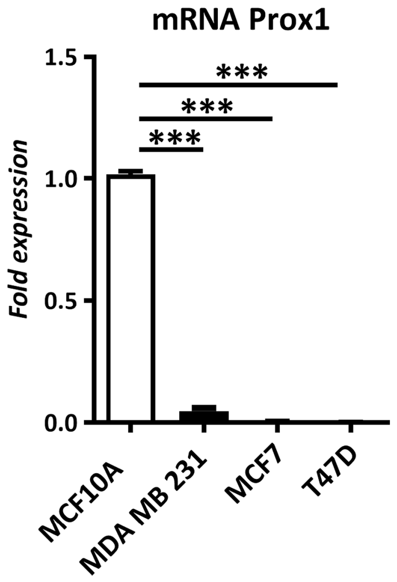

B

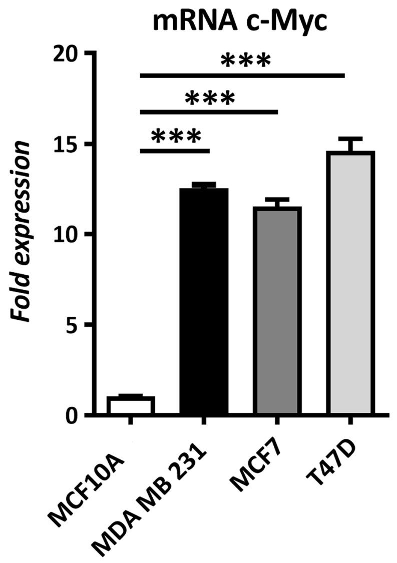

Supplementary Figure S2. Prox1 and c-Myc expression in various breast cell lines. (A) Relative expression levels of Prox1 in MCF10A, MDA-MB-231, MCF7, and T47D cells, measured with quantitative real time RT-PCR. (B) Relative expression levels of c-Myc in

MCF10A, MDA-MB-231, MCF7 and T47D cells, measured with quantitative real time RT-PCR. For all cases, nst=p>0.05, *p<0.05, **p<0.01, ***p<0.001.

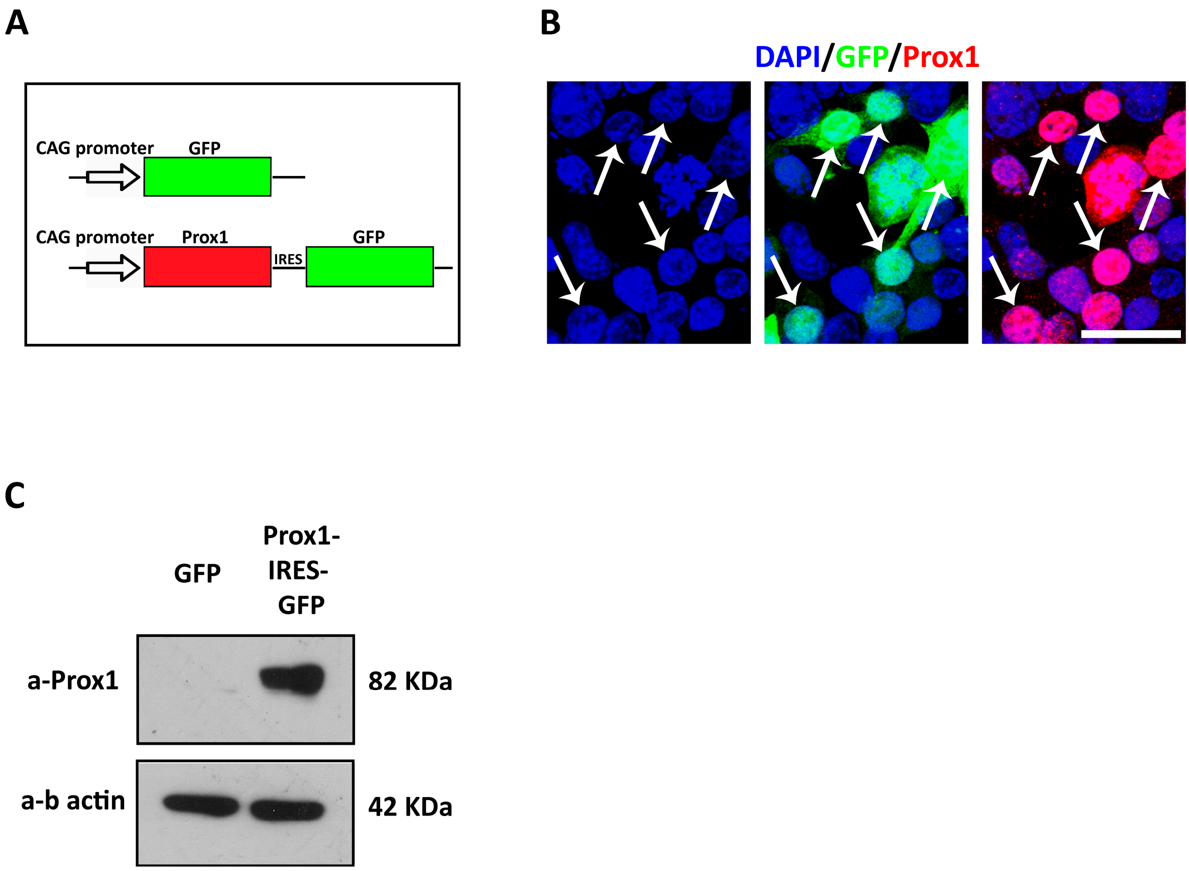

Supplementary Figure S3. Confirmation of Prox1 over-expression with Prox1-IRES-GFP plasmid. (A) Schematic representation of the two expression vectors: pCAGGs-GFP and pCAGGs-Prox1-IRES-GFP used in this study. (B) HEK293A cells were transfected with pCAGGs-GFP or pCAGGs-Prox1-IRES-GFP plasmids and immunostained for GFP (green) and Prox1 protein (red). Note that in all cases GFP positive cells are always Prox1 positive. Scale bar: 100 μm. (C) HEK293A cells were transfected with pCAGGs-GFP or pCAGGs-

Prox1-IRES-GFP plasmids. Western blot analysis of Prox1 and b-actin in HEK293A cells in pCAGGs-GFP and pCAGGs-Prox1-IRES-GFP over-expression conditions.

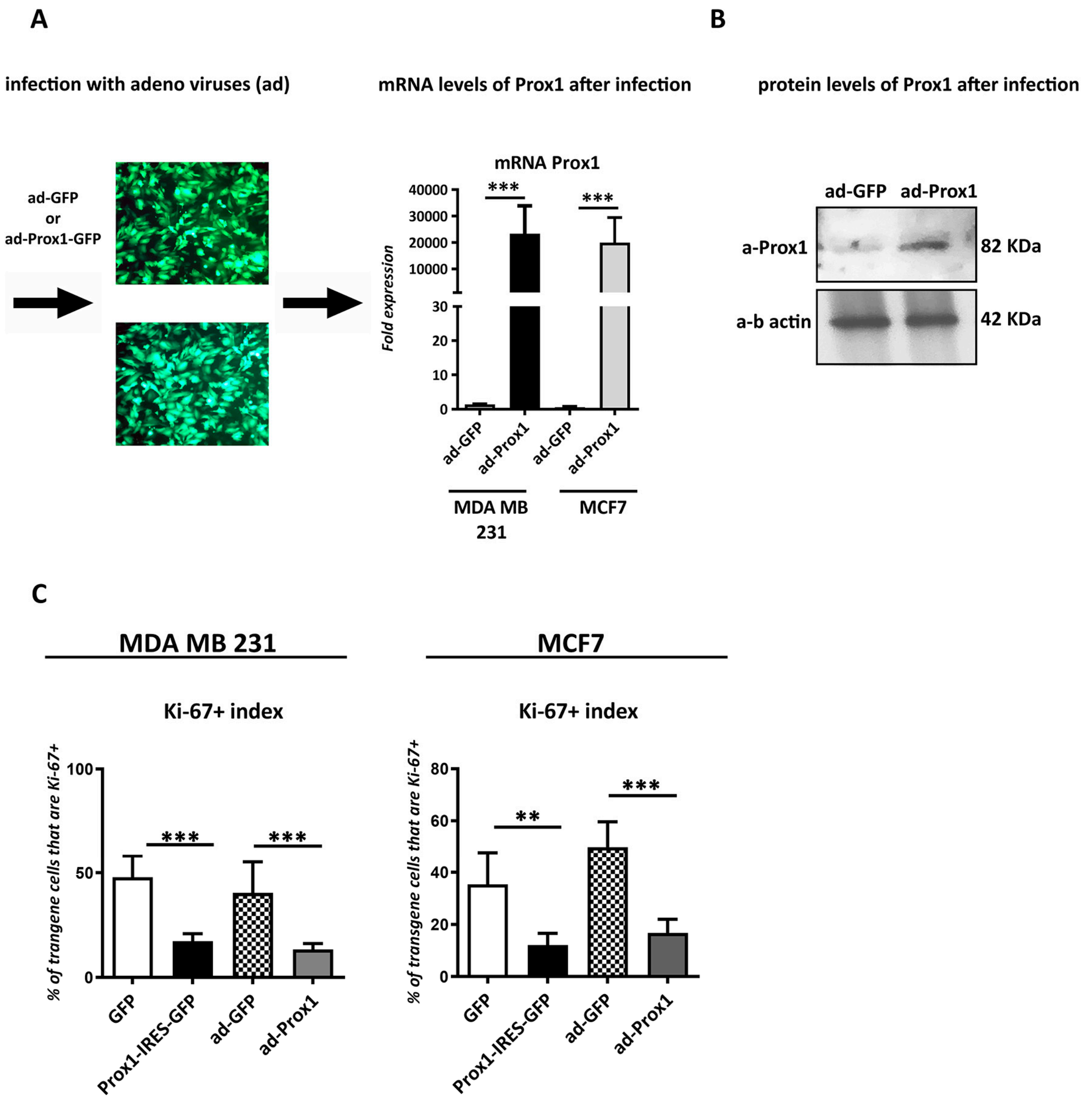

Supplementary Figure S4. Confirmation of Prox1 over-expression with the adenoviral system. (A) Representation of the experimental expression strategy which shows the over-expression with adeno-GFP virus or with adeno-Prox1-GFP virus. Relative expression levels of Prox1 in MDA-MB-231 and MCF7 cells, in ad-GFP or ad-Prox1 over-expression conditions measured with quantitative real time RT-PCR. (B) Western blot analysis of Prox1 and b-actin

in MCF7 cells in ad-GFP and ad-Prox1 over-expression conditions. (C) Quantification of Ki-67-positive cells in transgene-positive MDA-MB-231 cells (GFP: 47.88 ± 4.395% vs Prox1-IRES-GFP: 17.34 ± 2,765%, $p<0.01$) and quantification of Ki-67 positive cells in adeno-viruses GFP and Prox1 over-expression conditions in MDA-MB-231 cells (ad-GFP: 40,37 ± 5,712% vs ad-Prox1: 13,35 ± 1,445%, $p<0.01$). Quantification of Ki-67-positive cells in transgene-positive MCF7 cells (GFP: 35,40 ± 5.818% vs Prox1-IRES-GFP: 12.09 ± 3.313%, $p<0.01$) and quantification of Ki-67 positive cells in adeno-viruses GFP and Prox1 over-expression conditions in MCF7 cells (ad-GFP: 49.08 ± 4.916% vs ad-Prox1: 14,08 ± 2.570%, $p<0.01$). For all cases, ns=$p>0.05$, *$p<0.05$, **$p<0.01$, ***$p<0.001$.

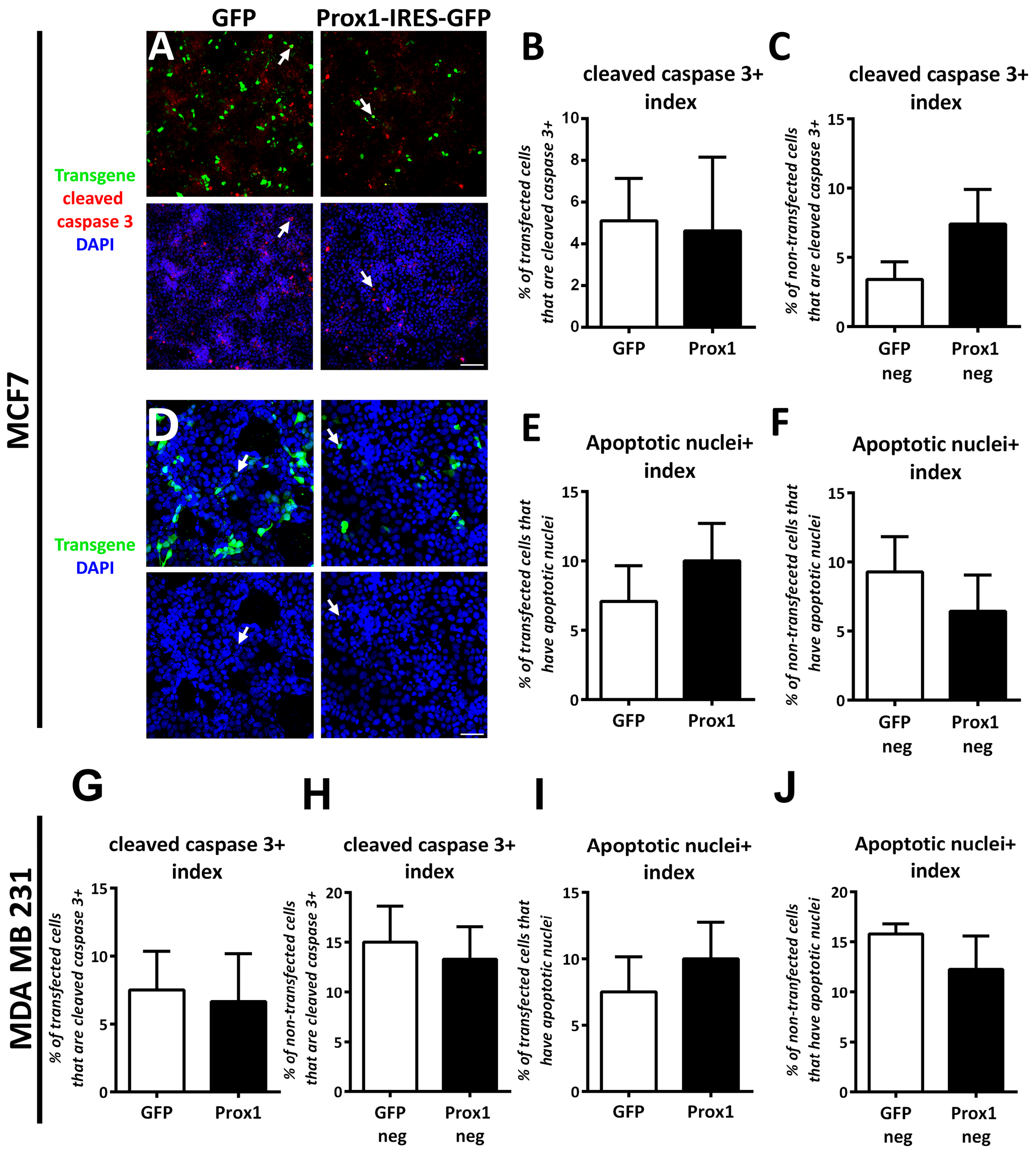

Supplementary Figure S5. Prox1 over-expression does not affect apoptosis of human breast cancer cells. (A) Prox1 and GFP transfected MCF7 cells were stained for cleaved caspase 3 (red) and labeled with DAPI (blue). Arrowheads indicate representative double positive cells (GFP positive and cleaved caspase positive or Prox1 positive and cleaved caspase positive).

Scale bar: 250 μM (B) Quantification of cleaved caspase 3-positive cells in transgene-positive MCF7 cells (GFP: 5.106383 ± 2.034432% vs Prox1: 4.62963 ± 3.531343%, p>0.1) (C) Quantification of cleaved caspase 3-positive cells in transgene-negative MCF7 cells (GFP: 3.404255 ± 1.286688% vs Prox1: 7.407407 ± 2.497037%, p>0.05). (D) Prox1 and GFP transfected MCF7 cells were measured for apoptotic nuclei and labeled with DAPI. Arrowheads indicate representative double positive cells (GFP positive and apoptotic nucleus positive or Prox1 positive and apoptotic nucleus positive). Scale bar: 75 μM. (E) Quantification of apoptotic nuclei-positive cells in transgene-positive MCF7 cells (GFP: 7.083333 ± 2.579295% vs Prox1: 10 ± 2.7024%, p>0.1) (F) Quantification of apoptotic nuclei-positive cells in transgene-negative MCF7 cells (GFP: 9.285714 ± 2.562419% vs Prox1: 6.428571 ± 2.640871%, p>0.1). (G) Quantification of cleaved caspase 3-positive cells in transgene-positive MDA-MB-231 cells (GFP: 7.5 ± 2.866328% vs Prox1: 6.666667 ± 3.531343%, p>0.1). (H) Quantification of cleaved caspase 3-positive cells in transgene-negative MDA-MB-231 cells (GFP: 15 ± 3.662842% vs Prox1: 13.33333 ± 3.213714%, p>0.1). (I) Quantification of apoptotic nuclei-positive cells in transgene-positive MDA-MB-231 cells (GFP: 7.5 ± 2.659148% vs Prox1: 10 ± 2.749416%, p>0.1). (J) Quantification of apoptotic nuclei-positive cells in transgene-negative MDA-MB-231 cells (GFP: 15.78947 ± 1% vs Prox1: 12.2807 ± 3.292507%, p>0.1). For all cases, ns (non-significant) for p>0.05, *p<0.05, **p<0.01, ***p<0.001.

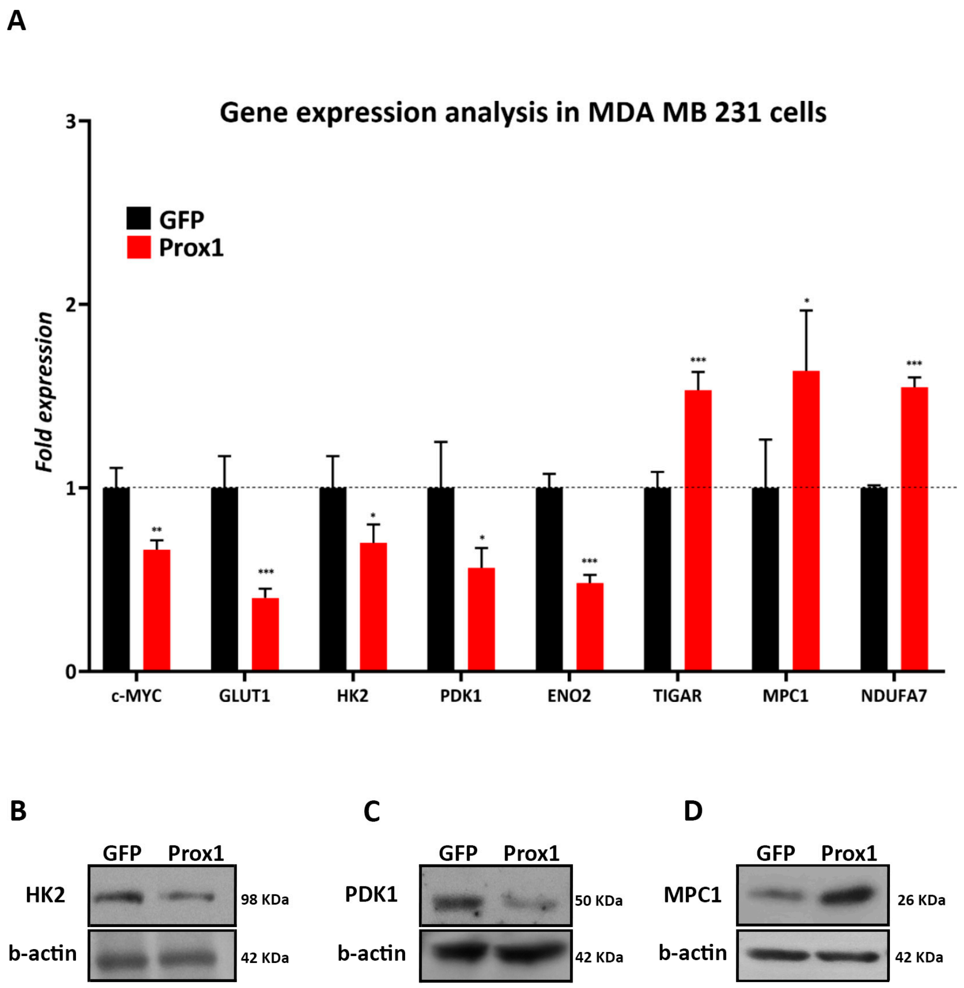

Supplementary Figure S6. Prox1 over-expression affects crucial regulators of glycolysis in MDA-MB-231 cells. (A) mRNA expression analysis of genes critically involved in tumor development in MDA-MB-231 cells over-expressing GFP or Prox1. Relative expression levels of c-MYC, GLUT1, HK2, PDK1, ENO2, TIGAR, MPC1 and NDUFA7 mRNA in GFP and Prox1 over-expression conditions, measured with quantitative real time RT-PCR. For all cases, ns=p>0.05, *p<0.05, **p<0.01, ***p<0.001. (B) Western blot analysis of HK2 and b-actin in MDA-MB-231 cells in GFP and Prox1 over-expression conditions. (C) Western blot analysis of

PDK1 and b-actin in MDA-MB-231 cells in GFP and Prox1 over-expression conditions. (D) Western blot analysis of MPC1 and b-actin in MDA-MB-231 cells in GFP and Prox1 over-expression conditions.

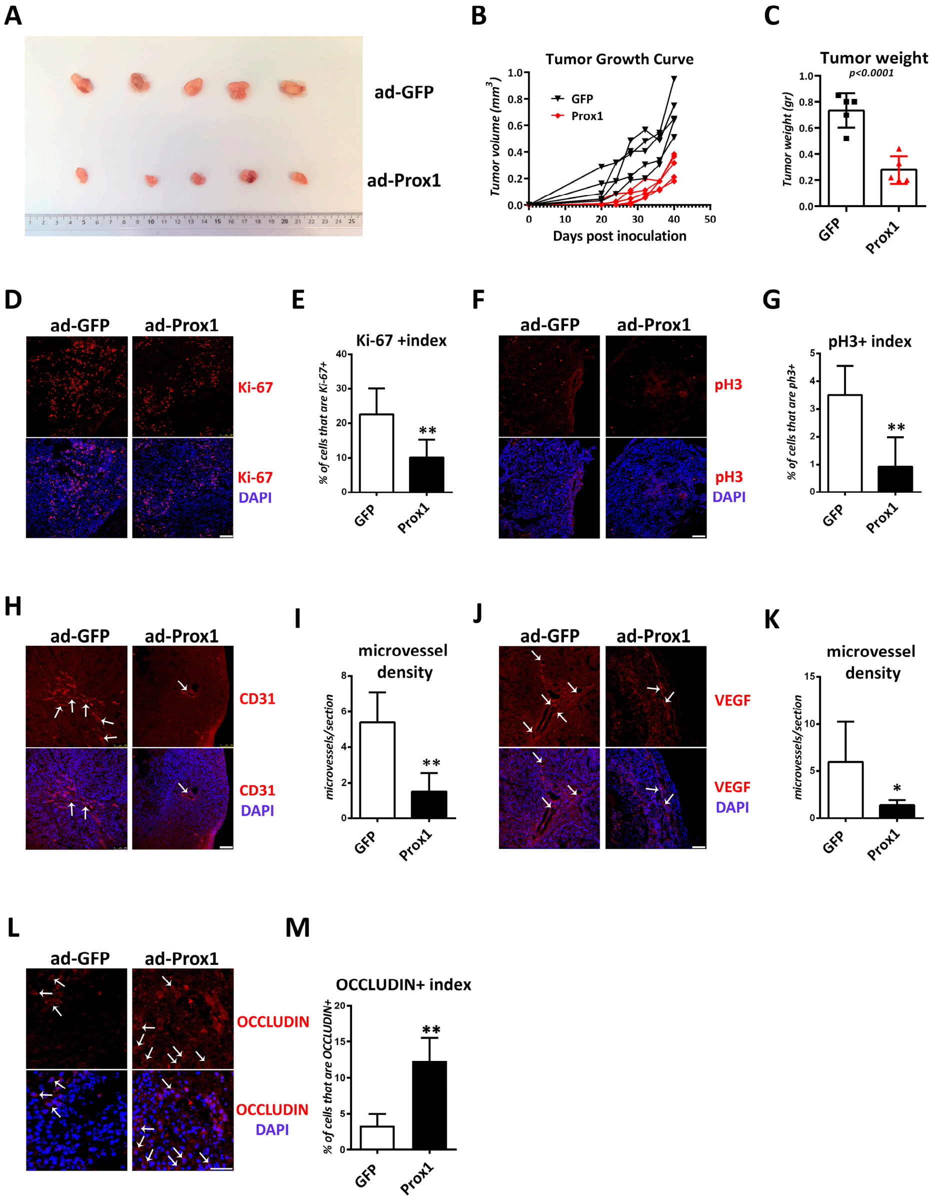

Supplementary Figure S7. Prox1 inhibits proliferation of human breast cancer cells *in vivo*. (A) Representative images of whole tumors that were grown in NOD/SCID animals after subcutaneous transplantation of MDA-MB-231 cells over-expressing GFP or Prox1. (B) Quantification of the tumor growth after the heterotopic injections of MDA-MB-231 cells over-expressing GFP and Prox1, as indicated. (C) Quantification of the tumor weight of the tumors over-expressing GFP and Prox1 (GFP: 0.7340 ± 0.1315 g vs Prox1: 0.2780 ± 0.1055 g, $p<0.001$). (D) Tumor sections of MDA-MB-231 cells over-expressing GFP and Prox1 were labeled for Ki-67 (red) and DAPI (blue). Tumors were collected at the end of the experiment. Scale bar: 40 μM. (E) Quantification of the Ki-67 index in GFP and Prox1 treated tumors (GFP: 22.55 ± 7.502% vs Prox1: 10.12 ± 5.098%, $p<0.01$). (F) Tumor sections of MDA-MB-231 cells over-expressing GFP and Prox1 were labeled for pH3 (red) and DAPI (blue). Tumors were collected at the end of the experiment. Scale bar: 40 μM. (G) Quantification of the pH3 index in GFP and Prox1 treated tumors (GFP: 3.500 ± 1.046% vs Prox1: 0.9074 ± 1.080%, $p<0.01$). (H) Tumor sections of MDA-MB-231 cells over-expressing GFP and Prox1 were labeled for CD31 (red) and DAPI (blue). Tumors were collected at the end of the experiment. Scale bar: 40 μM. (I) Quantification of the microvessel density in GFP and Prox1 treated tumors (GFP: 5.400 ± 1.673% vs Prox1: 1.500 ± 1.049%, $p<0.01$). (J) Tumor sections of MDA-MB-231 cells over-expressing GFP and Prox1 were labeled for VEGF (red) and DAPI (blue). Tumors were collected at the end of the experiment. Scale bar: 40 μM. (K) Quantification of the of the microvessel density in GFP and Prox1 treated tumors (GFP: 6.000 ± 4.243% vs Prox1: 1.400 ± 0.5477%, $p<0.05$). (L) Tumor sections of MDA-MB-231 cells over-expressing GFP and Prox1 were labeled for OCCLUDIN (red) and DAPI (blue). Tumors were collected at the end of the experiment. Scale bar: 75 μM. (M) Quantification of OCCLUDIN-positive cells over all cells in GFP and Prox1 treated tumors (GFP: 3.333 ± 1.667% vs Prox1: 12.35 ± 3.152%, $p<0.01$). For all cases, ns (non-significant) for $p>0.05$, *$p<0.05$, **$p<0.01$, ***$p<0.001$.

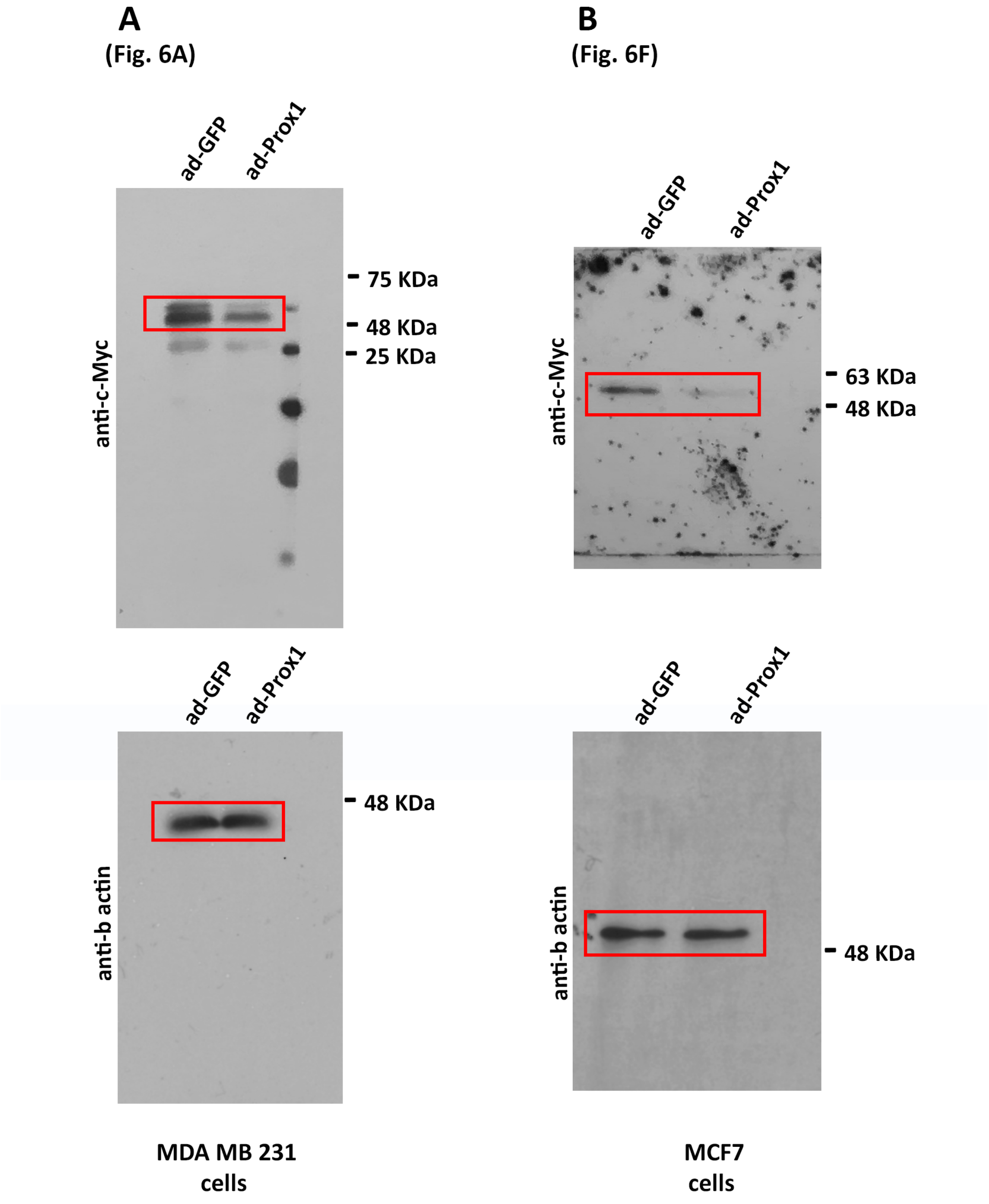

Supplementary Figure S8. Original full-size scans of the western blots presented in Figure 6. (A) Original scans of the western blot shown in Figure 6A. (B) Original scans of the western blot shown in Figure 6F.

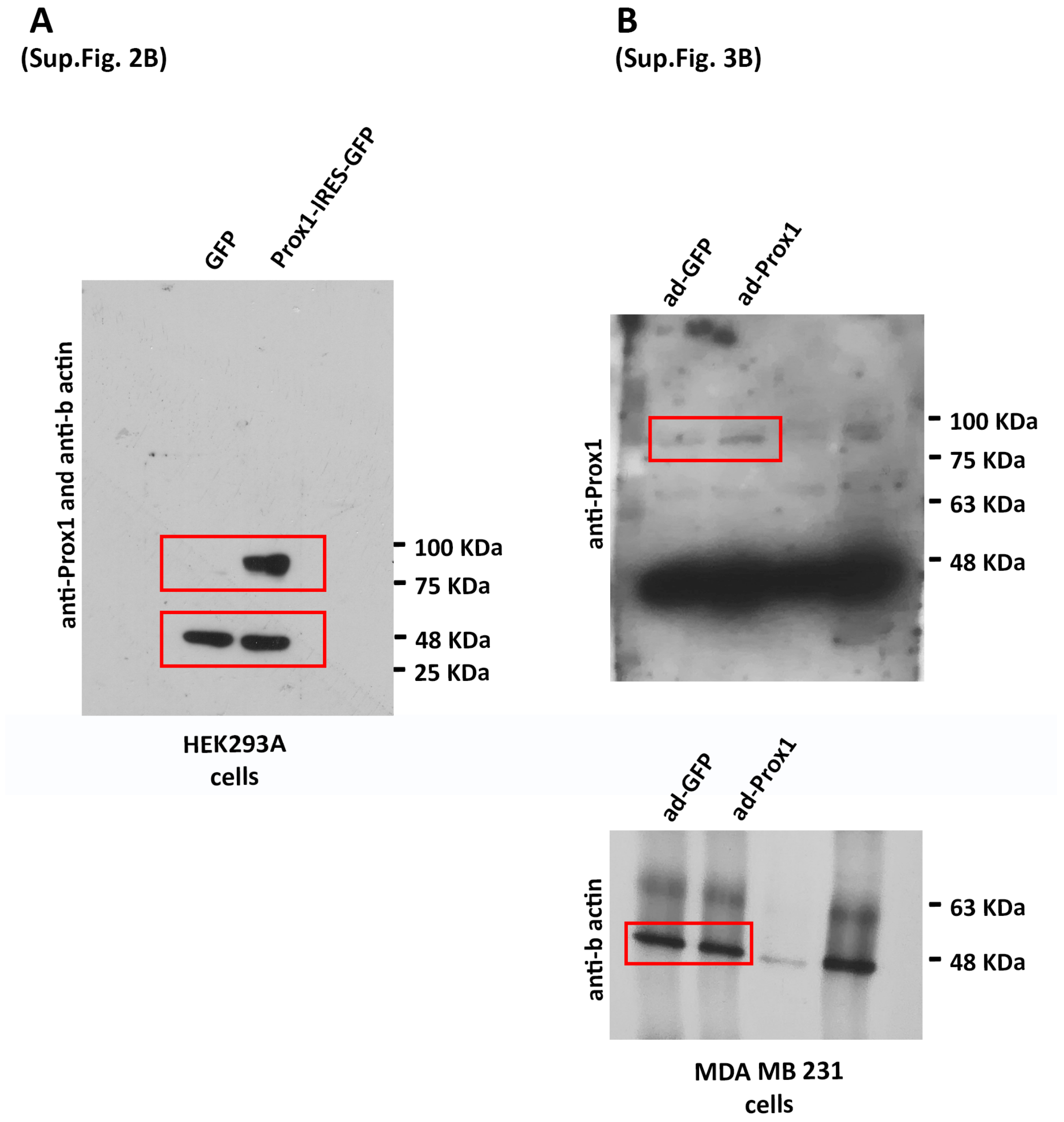

Supplementary Figure S9. Original full-size scans of the western blots presented in Supplementary Figure S2 and Supplementary Figure S3. (A) Original scans of the western blot shown in Supplementary Figure S2B. (B) Original scans of the western blot shown in Supplementary Figure S3B.

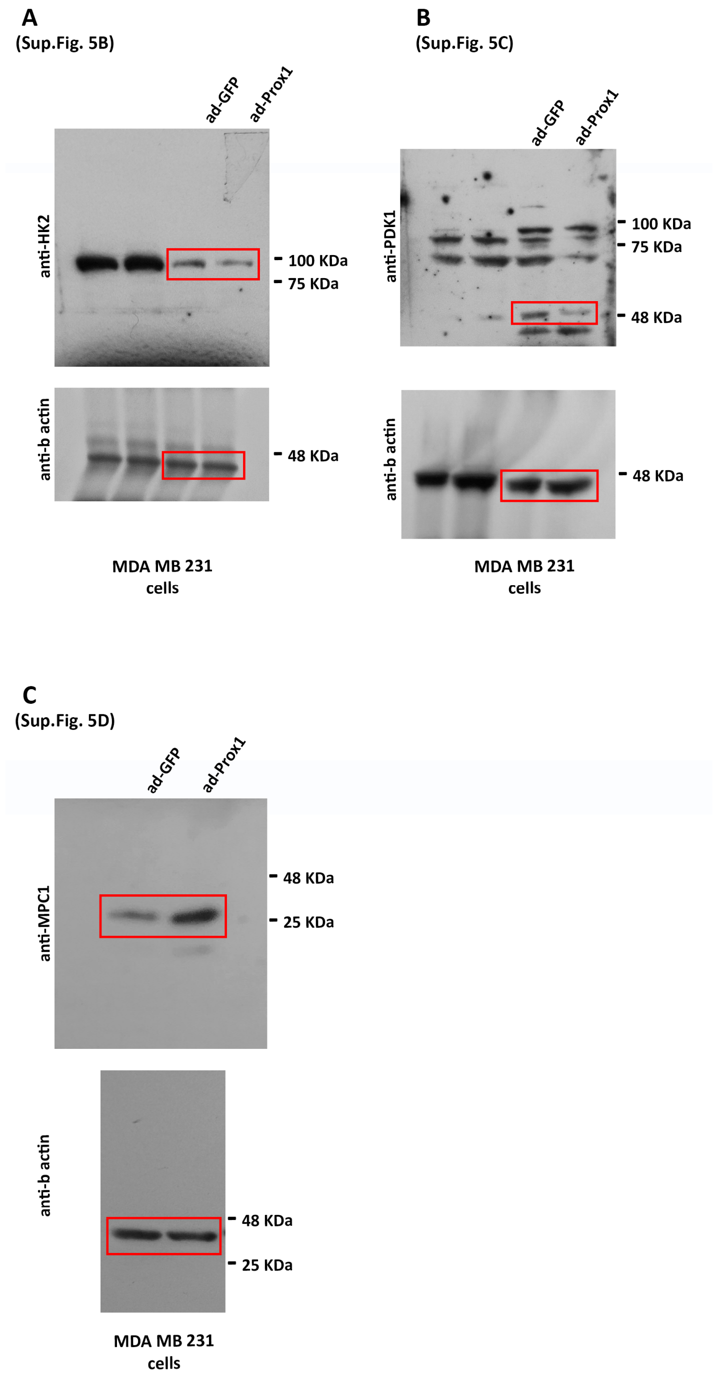

Supplementary Figure S10. Original full-size scans of the western blots presented in Supplementary Figure S5. (A) Original scans of the western blot shown in Supplementary

Figure S5B. (B) Original scans of the western blot shown in Supplementary Figure S5C. (C) Original scans of the western blot shown in Supplementary Figure S5D.
